# Improved safety and efficacy of ^213^Bi-DOTATATE-targeted alpha therapy of somatostatin receptor-expressing neuroendocrine tumors in mice pre-treated with l-lysine

**DOI:** 10.1186/s13550-016-0240-5

**Published:** 2016-11-21

**Authors:** Ho Sze Chan, Mark W. Konijnenberg, Tamara Daniels, Monique Nysus, Mehran Makvandi, Erik de Blois, Wouter A. Breeman, Robert W. Atcher, Marion de Jong, Jeffrey P. Norenberg

**Affiliations:** 1 Department of Radiology and Nuclear Medicine, Erasmus MC, ‘s Gravendijkwal 230, 3015 CE Rotterdam, The Netherlands; 2 Radiopharmaceutical Sciences Program, College of Pharmacy, University of New Mexico Health Sciences Center, Albuquerque, NM USA; 3 Los Alamos National Laboratory, Los Alamos, NM USA

**Keywords:** Targeted alpha therapy, ^213^Bi-DOTATATE, Nephrotoxicity, Maximum tolerated dose, l-lysine

## Abstract

**Background:**

Targeted alpha therapy (TAT) offers advantages over current β-emitting conjugates for peptide receptor radionuclide therapy (PRRT) of neuroendocrine tumors. PRRT with ^177^Lu-DOTATATE or ^90^Y-DOTATOC has shown dose-limiting nephrotoxicity due to radiopeptide retention in the proximal tubules. Pharmacological protection can reduce renal uptake of radiopeptides, e.g., positively charged amino acids, to saturate in the proximal tubules, thereby enabling higher radioactivity to be safely administered. The aim of this preclinical study was to evaluate the therapeutic effect of ^213^Bi-DOTATATE with and without renal protection using L-lysine in mice. Tumor uptake and kinetics as a function of injected mass of peptide (range 0.03–3 nmol) were investigated using ^111^In-DOTATATE. These results allowed estimation of the mean radiation absorbed tumor dose for ^213^Bi-DOTATATE. Pharmacokinetics and dosimetry of ^213^Bi-DOTATATE was determined in mice, in combination with renal protection. A dose escalation study with ^213^Bi-DOTATATE was performed to determine the maximum tolerated dose (MTD) with and without pre-administration of l-lysine as for renal protection. Neutrophil gelatinase-associated lipocalin (NGAL) served as renal biomarker to determine kidney injury.

**Results:**

The maximum mean radiation absorbed tumor dose occurred at 0.03 nmol and the minimum at 3 nmol. Similar mean radiation absorbed tumor doses were determined for 0.1 and 0.3 nmol with a mean radiation absorbed dose of approximately 0.5 Gy/MBq ^213^Bi-DOTATATE. The optimal mass of injected peptide was found to be 0.3 nmol. Tumor uptake was similar for ^111^In-DOTATATE and ^213^Bi-DOTATATE at 0.3 nmol peptide. Lysine reduced the renal uptake of ^213^Bi-DOTATATE by 50% with no effect on the tumor uptake. The MTD was <13.0 ± 1.6 MBq in absence of l-lysine and 21.7 ± 1.9 MBq with l-lysine renal protection, both imparting an LD_50_ mean renal radiation absorbed dose of 20 Gy. A correlation was found between the amount of injected radioactivity and NGAL levels.

**Conclusions:**

The therapeutic potential of ^213^Bi-DOTATATE was illustrated by significantly decreased tumor burden and improved overall survival. Renal protection with l-lysine immediately prior to TAT with ^213^Bi-DOTATATE prolonged survival providing substantial evidence for pharmacological nephron blockade to mitigate nephrotoxicity.

**Electronic supplementary material:**

The online version of this article (doi:10.1186/s13550-016-0240-5) contains supplementary material, which is available to authorized users.

## Background

Targeted alpha therapy (TAT) has shown great promise in the treatment of both micrometastatic [1] and large solid tumors in preclinical and clinical studies [2, 3]. Alpha-emitters emit high linear energy transfer (LET) α-particles, each causing dense ion pairs (2000–7000) within a relative short path length (50–100 μm) [3]. The radioactive decay of ^213^bismuth (^213^Bi, *T*_1/2_ = 46 min) results in the emission of high-LET α-particles by ^213^Bi self and by its daughter ^213^Po around 100 keV/μm. Due to the relative short half-life of ^213^Bi, ^213^Bi can deliver a high radiation dose rate to the target within a relatively short period of time. These physical characteristics make ^213^Bi, one of the most commonly used α-emitters for medical applications, with demonstrated promise as TAT in preclinical studies, in vivo imaging, and in clinical treatment of cancer patients [1, 4, 5].

Peptide receptor radionuclide therapy (PRRT) with radiolabeled somatostatin analogs is commonly employed in patients with inoperable neuroendocrine tumors (NETs) overexpressing somatostatin receptors subtype 2 (SSTR_2_). Current radiopeptides include ^177^Lu-[DOTA^0^,Tyr^3^]octreotate (^177^Lu-DOTATATE) and ^90^Y-[DOTA^0^,Tyr^3^]octreotide (^90^Y-DOTATOC). Its efficacy depends on the radiation absorbed dose delivered to the tumor, which depends on SSTR_2_ targeting efficiency, clearance kinetics, perfusion, distribution, and tumor mass. High-specific activity radiopeptides are required to deliver adequate radiation absorbed dose to tumors, as the mass of injected peptide is limited by the high affinity but low capacity of SSTR_2_-expression systems. The mass of injected peptide influences the pharmacokinetics (PK) and absorbed doses in organs and tumors [6]. Therefore, the mass of injected peptide should be optimized to deliver efficacious tumor doses while avoiding toxic absorbed dose to organs, especially to the dose-limiting organs the kidneys and bone marrow [7, 8].

Radiolabeled somatostatin analogs are known to accumulate in the renal proximal tubules, due to their net charge, electrostatic forces, and charge distribution from metal-chelation [9, 10]. This can result in a high absorbed dose and subsequent renal dysfunction. Co-infusion of l-lysine/l-arginine has been shown to reduce renal uptake in patients receiving ^177^Lu-DOTATATE or ^177^Lu-DOTATOC PRRT by 30–50% [11].

Several preclinical studies showed that TAT with ^213^Bi resulted in high renal accumulation of radioactivity [12], causing nephrotoxicity and decreased survival without renal protection compared to animals receiving protection [13, 14]. Evidence of acute or chronic interstitial nephritis was found in a previous dose escalation study in AR42J tumor-bearing rats using ^213^Bi-DOTATOC [2]. Nephrotoxicity was observed to be moderate in a clinical trial of ^213^Bi-DOTATOC, in combination with renal protection, in patients’ refractory to ^177^Lu-DOTATATE or ^90^Y-DOTATOC PRRT [3]. Conventional approaches to determine kidney function use serum creatinine or nuclear medicine imaging with ^99m^Tc-MAG3 or ^99m^Tc-DSMA. However, these approaches are suboptimal to detect early-stage kidney disease. Several renal biomarkers are commercially available to determine acute or chronic kidney injury [15]. However, those biomarkers have not yet been applied in PRRT for detection of nephrotoxicity. Neutrophil gelatinase-associated lipocalin (NGAL) is among the promising renal biomarkers for detection of acute or chronic kidney injury in humans with high specificity and sensitivity [16]. Therefore, NGAL is an interesting renal biomarker to study nephrotoxicity caused by TAT with ^213^Bi.

This study aimed to determine the suitability of ^213^Bi-DOTATATE for TAT. Administration of ^213^Bi-DOTATATE was optimized for in vivo applications in AR42J tumor-bearing mice. The rat AR42J tumor is known to express SSTR_2_ at high density; this model is commonly used for investigations using somatostatin analogs and PRRT. Additionally, investigations were performed on increasing ^213^Bi-DOTATATE’s efficacy by using l-lysine as a renal protectant, radiation dosimetry to determine the mean radiation absorbed dose to the tumor and kidney, the resultant dose-effect relation, and a pilot study to evaluate NGAL as a kidney injury biomarker.

## Methods

### ^213^Bi-DOTATATE labeling

^213^Bi was eluted from a ^225^Ac/^213^Bi generator (Oak Ridge National Laboratory) with 0.1 M/0.1 M HCl/NaI. The resultant elution containing ^213^Bi (630–740 MBq) was used for labeling with 10 μg DOTATATE (BioSynthema) in a reaction vial including 0.15 M TRIS buffer and 2.6 mM ascorbic acid at pH 8.4. The reaction was incubated for 5 min at 95 °C and cooled to ambient temperature for 2 min before adding 50 mM DTPA [17]. Instant thin-layer chromatography (ITLC-SG, Varian) was performed using 0.9% NaCl as mobile phase to determine the radionuclide-peptide incorporation yield. High-performance liquid chromatography (HPLC, Agilent) was performed to determine the radiochemical purity (RCP) of ^213^Bi-DOTATATE, being defined as percentage of intact radiopeptide of interest compared to other detectable radioactive compounds in the same HPLC analysis. HPLC was performed using a reverse phase C_18_ column (JT Baker, Bakerbond®, 4.6 × 250 mm) eluted with 0.1% TFA and methanol [18].

### ^111^In-DOTATATE labeling

^111^InCl_3_ (GE Healthcare) was added to a vial containing 0.03, 0.1, 0.3, 1, or 3 nmol DOTATATE. ^115^In(NO_3_)_3_ (0.01 g/L, ICP standard) was added to form a 1:1 M ratio reaction to peptide, and NaOAc 4 M was used to adjust the pH to 4–5. The reaction was heated at 80 °C for 20 min and cooled to ambient temperature for 5 min before the addition of DTPA (50 mM) to incorporate potential free ^111^In^3+^. Incorporation yields of the labeled peptide were evaluated as described previously.

### Animal model

Athymic male nu/nu mice (Tachonic), 6–8 weeks old, were used in all studies. Tumor models were established by inoculating 5 × 10^6^ rat pancreatic tumor AR42J cells (American Type Culture Collection) with high SSTR_2_ expression into the right hind flank of the animals. After 3 weeks, the tumor size reached approximately 200 mm^3^. All animal experiments were carried out following Institutional Animal Care and Use Committee-approved protocol.

### Comparison of biodistribution profiles of ^111^In-DOTATATE and ^213^Bi-DOTATATE

AR42J tumor-bearing animals were used for the comparison of the uptake of ^111^In-DOTATATE versus (vs.) ^213^Bi-DOTATATE in different organs and tumors. Biodistribution assays were performed with either ^111^In-DOTATATE or ^213^Bi-DOTATATE (0.3 nmol, *n* = 3/cohort). Animals were euthanized 10 and 60 min post-injection (p.i.) by CO_2_ asphyxiation. Blood samples were collected, and the following organs were harvested and counted in a γ-counter (PerkinElmer): tumor, blood, heart, adrenals, kidneys, stomach without content, pancreas, liver, testicles, urinary bladder, femur, femur marrow, pituitary, and muscles. The uptake was expressed as percentage of injected activity per gram of tissue (%IA/g). The actual weight of all organs was used to calculate %IA/g.

### Biodistribution ^111^In-DOTATATE

Xenograft AR42J nu/nu mice were used to determine PK as a function of injected mass of peptide (*n* = 4/cohort). Animals were injected intravenously (i.v.) via the tail vein with 0.03, 0.1, 0.3, 1, or 3 nmol (corresponding to 2 × 10^−3^, 7 × 10^−3^, 0.02, 0.07, and 0.22 mg/kg, respectively) of ^111^In-DOTATATE (range 0.6–2.9 MBq). Animals were euthanized by CO_2_ asphyxiation at 3, 10, 30, and 60 min p.i. Blood samples, organs, and femur-containing femur marrow were harvested and counted as described previously. The uptake was expressed as percentage of injected activity per gram of tissue (%IA/g).

### The effect of l-lysine on the biodistribution of ^213^Bi-DOTATATE in tumor and nontumor-bearing mice

Twenty AR42J tumor-bearing mice, 200 mm^3^ tumor volume, were injected intraperitoneally (i.p.) with or without (w/wo) 35 mg/200 μL l-lysine (l-lysine monohydrochloride) 2–10 min prior to ^213^Bi-DOTATATE administration i.v. (1–3 MBq/0.3 nmol). Mice were euthanized at 10 and 60 min p.i. In a parallel study, 20 nontumor-bearing mice were injected i.p. w/wo l-lysine (35 mg/200 μL) 2–10 min prior to ^213^Bi-DOTATATE (1–3 MBq/0.3 nmol) administration and were euthanized at 10 and 60 min p.i (*n* = 5/cohort). Blood, organs, and femur-containing femur marrow were collected and counted as described previously. The uptake was expressed as percentage of injected activity per gram of tissue (%IA/g).

### Toxicity and therapeutic efficacy of ^213^Bi-DOTATATE

Toxicity and therapeutic efficacy were investigated in 18 animals (*n* = 6/cohort): control, low-dose (cumulative 16.8 ± 1.3 MBq), and high-dose (cumulative 33.1 ± 3.7 MBq) cohorts. Treatment started 23 days after xenograft inoculation. Control mice received in total 4 × 0.3 nmol DOTATATE on two consecutive days. Two injections of 0.3 nmol DOTATATE per day were given, with a time interval of at least 6 h. The low-dose cohort received two doses of ^213^Bi-DOTATATE on two consecutive days, one dose of 8.3 ± 1.0 MBq/0.3 nmol ^213^Bi-DOTATATE each day. The high-dose cohort received four doses on two consecutive days, twice a day with a time interval of at least 6 h. Per dose, ^213^Bi-DOTATATE contained 8.3 ± 1.0 MBq/0.3 nmol ^213^Bi-DOTATATE. Animals were monitored for 30 days starting on the day of treatment. Tumors were measured by caliper, and animals were weighed at least twice weekly. The endpoints chosen were weight loss >15% and tumor volume >2000 mm^3^. At 30 days post-treatment, blood samples were obtained for hematological analysis according to the standard operating procedures for clinical laboratory samples for creatinine, CBC, WBC, RBC, Hgb, Hct, MCV, MCHC, platelets, neutrophils, lymphocytes, monocytes, eosinophils, and basophils. Survival analysis was plotted according to the Kaplan-Meier fit model.

Tumor volume *V*(*t*) as a function of time was modeled according to the exponential growth function *V*(*t*) = *V*_0_ × *e* ^*kt*^, with *k* the growth constant, related to the doubling time *T*_*d*_ by $k=\frac{ \ln (2)}{T_d}$. Each individual mouse *V*(*t*) in the control group was fitted with the exponential growth function to enable extrapolation of the growth beyond the time when the tumor volume exceeded the maximum. An average control growth curve was obtained by using the mean of the volume data together with the extrapolated growth data to the time points of the last surviving animal. Fitting was also performed for the therapy group with an exponential growth function, where the initial growth rate *k*_0_ slowed down or turned into shrinkage with rate *k*_0_ − *k*_1_ at onset time point *T*_0_ of therapy effect. Regrowth was modeled by exponential growth with rate *k*_0_ *− k*_1_ *+ k*_2_, setting in after the volume nadir time point *T*_1_. This led to the function $V(t)={V}_0\times {e}^{k_0t}\times { \max}_{t>{T}_1}e{}^{-{k}_1\left(t-{T}_1\right)}\times { \max}_{t>{T}_2}{e}^{k_2\left(t-{T}_2\right)}$. The regrowth doubling time *T*_rd_ was derived from ${T}_{\mathrm{rd}}=\frac{ \ln (2)}{k_0-{k}_1+{k}_2}$.

### Maximum tolerated dose (MTD) of ^213^Bi-DOTATATE in nontumor-bearing mice in combination of l-lysine

MTD was defined as the highest dose given to the animals allowing 100% survival with no significant weight loss >15% throughout the experiment. Nontumor-bearing mice were randomly divided into seven cohorts used to evaluate MTD, six treatments and one control (*n* = 8/cohort); see Table 1. Cohorts(+) received i.p. injections of l-lysine (35 mg/200 μL) at 2–10 min prior to ^213^Bi-DOTATATE administration via i.v. tail vein. Control mice received DOTATATE (4 × 0.3 nmol) on four consecutive days. The animals were followed for 90 days. Serum was analyzed for the biomarker neutrophil gelatinase-associated lipocalin (NGAL) using ELISA (R&D Systems, 450 nm). Survival analysis was plotted according to the Kaplan-Meier fit model.

**Table 1:** ^213^Bi-DOTATATE toxicity study in nontumor-bearing animals with or without pre-injection of l-lysine (35 mg), *n* = 8/cohort

MTD study in nontumor-bearing animals in combination with l-lysine			
Cohort	^213^Bi-DOTATATE (MBq)	l-lysine	Days of injection
Low-dose(−)	13.0 ± 1.6	−	2
Low-dose(+)	13.2 ± 0.9	+	2
Medium-dose(−)	20.7 ± 0.8	−	3
Medium-dose(+)	21.7 ± 1.9	+	3
High-dose(−)	28.7 ± 1.2	−	4
High-dose(+)	28.3 ± 0.8	+	4
Control	0		4

*MTD* maximum tolerated dose

Control animals received 4 × 0.3 nmol DOTATATE (*n* = 8)

### Pharmacokinetics

Saturation of receptor-specific tumor uptake was investigated by determining the kinetics of the tumor uptake with increasing injected mass of peptide. The time-activity curves for the tumor and normal organs were fitted by single-exponential functions using Prism-5 (GraphPad). Goodness of fit was analyzed with the Pearson correlation coefficient *R* ^2^ > 0.8. Both *F* test and the Aikake information criterion were used to decide on the complexity of the curves.

### Radiation dosimetry

Cumulated radioactivity in the tumor and normal organs were estimated by integrating the time-activity curves fitted to the ^111^In-DOTATATE biodistribution data and folded with the decay curve of ^213^Bi and its daughters. Dosimetry was performed according to the MIRD schema by using the spherical nodes S-factors from the Olinda/EXM software [19]. S-factors were interpolated from the actual weight of the organs and tissue. All organs and tissue were assumed to be spherical with a density of 1 kg/m^3^. Mean radiation absorbed doses were obtained as a function-injected mass of peptide, assuming a homogeneous distribution in the tumor and organs. The mean radiation absorbed dose obtained included the cumulative dose of α, β, and γ from all daughters of ^213^Bi. Owing to the short path length of α-particles, only the self-dose within each organ was included. The threshold for lethality was determined with renal absorbed dose and injected activity as indicators. Logistic regression analysis was used to determine the LD_50_ for presumed renal toxicity-related death.

### Statistics

Data analyses, graphs, and calculations were performed in Prism-5. Mann-Whitney *t* test was used to calculate the significance. The results of statistical tests were considered significant when *P* was <0.05. Biodistribution data were expressed as mean ± standard deviation (SD) and tumor volume data as mean ± standard error (SEM). Binary logistic analysis (forced entry method) was performed with SPSS software (IBM SPSS statistics, version 20).

## Results

### Radiolabeling

The radiolabeling incorporation yield of ^213^Bi-DOTATATE was >95% and RCP was >85%; the incorporation yield of ^111^In-DOTATATE was >95%.

### Biodistribution of ^111^In-DOTATATE as function of injected mass of peptide and time

**Fig. 1:**
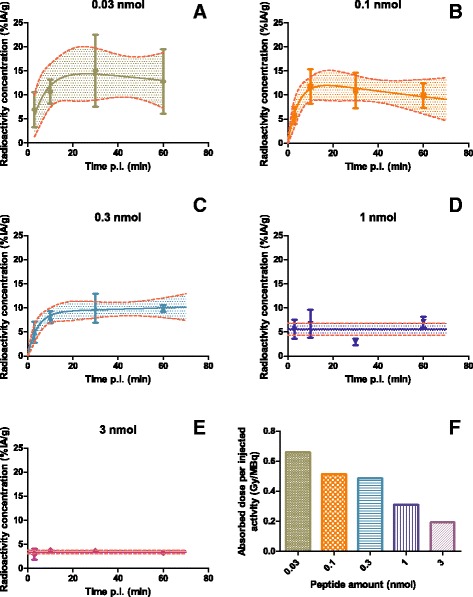
**a**–**e** Tumor uptake (%IA/g) of ^111^In-DOTATATE as function of time and injected mass of peptide (0.03–3 nmol, *n* = 4/cohort). The uptake was expressed as percentage of injected activity per gram of tissue (%IA/g). **f** Predicted absorbed tumor dose of ^213^Bi-DOTATATE based on ^111^In-DOTATATE tumor biodistribution results. The injected mass of peptide expressed on *x*-axis and the estimated mean radiation absorbed dose (Gy) per injected MBq ^213^Bi-DOTATATE on the *y*-axis

In animals injected with <0.3 nmol ^111^In-DOTATATE, higher tumor uptake was observed than in animals injected with >0.3 nmol, Fig. 1a–f. At lower injected mass of peptide (0.03, 0.1, and 0.3 nmol), tumor uptake increased as function of time. At >0.3 nmol of injected peptide, the uptake of ^111^In-DOTATATE was more uniform and low compared to <0.3 nmol of injected peptide, indicating the receptors on the tumors were partially saturated by the injected masses of unlabeled peptide. Renal uptake was higher compared to other organs for all injected mass of peptide at all time points, except for tumor. Organ uptake of 0.03–3 nmol mass of injected peptide are indicated in Additional file 1: Tables S2–S6.

Decreased absorbed tumor doses from ^213^Bi-DOTATATE as a function of increased of injected mass of peptide was predicted based on the ^111^In-DOTATATE uptake data. The mean radiation absorbed tumor dose ranged between 0.66 Gy/MBq ^213^Bi-DOTATATE at 0.03 nmol peptide and 0.19 Gy/MBq ^213^Bi-DOTATATE at 3 nmol peptide; see Fig. 1f. A comparable mean radiation absorbed dose of 0.50 Gy/MBq was found for 0.1 and 0.3 nmol peptide.

### Biodistribution of ^111^In-DOTATATE vs. ^213^Bi-DOTATATE

Tumor uptake of ^213^Bi-DOTATATE compared to ^111^In-DOTATATE was 5.3 ± 2.8%IA/g vs. 6.3 ± 1.3%IA/g at 10 min p.i., respectively (*P* = 0.70). Similar results were observed at 60 min p.i.: 6.5 ± 2.3%IA/g vs. 6.0 ± 1.2%IA/g, respectively (*P* = 1.0).

**Fig. 2:**
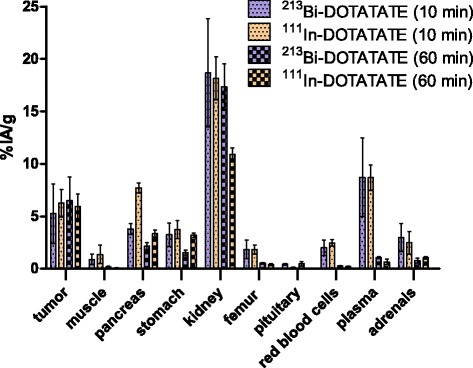
Comparative biodistributions (%IA/g ± SD) after i.v. administration of ^213^Bi-DOTATATE vs. ^111^In-DOTATATE (0.3 nmol peptide, *n* = 3/cohort) at 10 and 60 min p.i. in AR42J tumor-bearing mice. The uptake was expressed as percentage of injected activity per gram of tissue (%IA/g)

Renal activity at 60 min p.i. for ^213^Bi-DOTATATE was significantly higher than ^111^In-DOTATATE: 17.4 ± 2.2%IA/g and 10.9 ± 0.6%IA/g, respectively (*P* = 0.0022). ^111^In-DOTATATE was retained longer in the pancreas at 10 and 60 min p.i., whereas ^213^Bi-DOTATATE radioactivity in plasma was higher at 60 min p.i. and uptake in pituitary at 10 and 60 min p.i. All other organs showed similar uptakes for ^111^In-DOTATATE and ^213^Bi-DOTATATE at 10 min and 60 min p.i.; see Fig. 2.

### Biodistribution of ^213^Bi-DOTATATE; influence of l-lysine

**Fig. 3:**
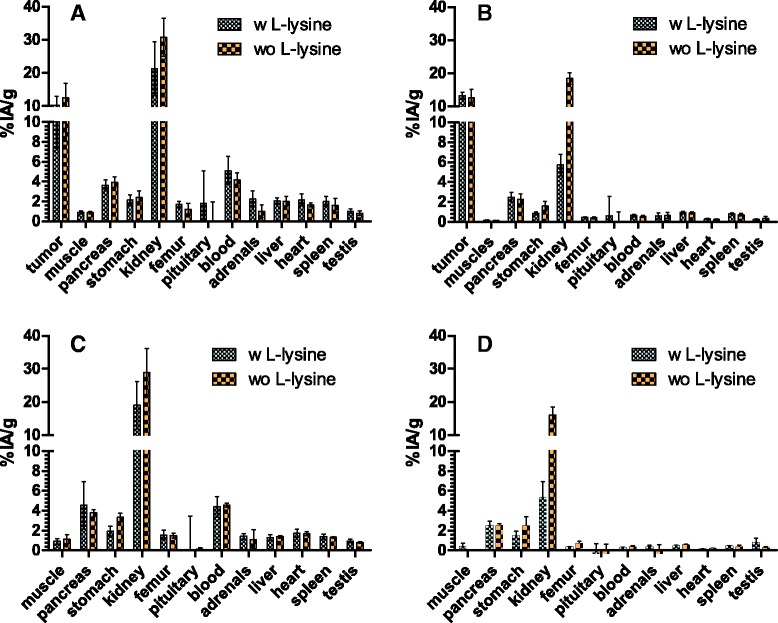
Biodistribution comparison of ^213^Bi-DOTATATE (0.3 nmol) with (w) or without (wo) l-lysine (35 mg) pre-injection. **a** AR42J tumor-bearing animals 10 min p.i. **b** AR42J tumor-bearing animals 60 min p.i. **c** Nontumor-bearing animals 10 min p.i. **d** Nontumor-bearing animals 60 min p.i., *n* = 5. The uptake was expressed as percentage of injected activity per gram of tissue (%IA/g)

Figure 3 shows the uptake of ^213^Bi-DOTATATE with or without l-lysine pre-injection in different organs and tissues in tumor-bearing and nontumor-bearing animals. Lower renal uptake was observed in tumor-bearing mice receiving l-lysine prior to administration of ^213^Bi-DOTATATE versus mice without l-lysine (10 min p.i.; 21.3 ± 8.1%IA/g vs. 30.8 ± 5.8%IA/g and 60 min p.i.; 5.7 ± 1.1%IA/g vs. 18.4 ± 1.8%IA/g (*P* <0.0001)).

No differences in tumor uptake were found in animals w/wo l-lysine pretreatment. However, at 60 min p.i., a significant difference in stomach uptake was found in tumor-bearing animals with and without l-lysine, 0.9 ± 0.1%IA/g versus 1.6 ± 0.5%IA/g, *P* = 0.0079. Figure 3 shows the uptake of ^213^Bi-DOTATATE w/wo l-lysine pre-injection in different organs in tumor- and nontumor-bearing animals.

In tumor-bearing animals receiving l-lysine, the renal absorbed dose was 0.56 Gy/MBq vs. 1.1 Gy/MBq without l-lysine. In nontumor-bearing animals, the renal absorbed dose was 0.50 Gy/MBq with l-lysine versus 1.0 Gy/MBq without l-lysine; see estimated mean radiation absorbed dose in Additional file 1: Table S7.

### Pharmacodynamics of ^213^Bi-DOTATATE in AR42J tumor-bearing animals

**Fig. 4:**
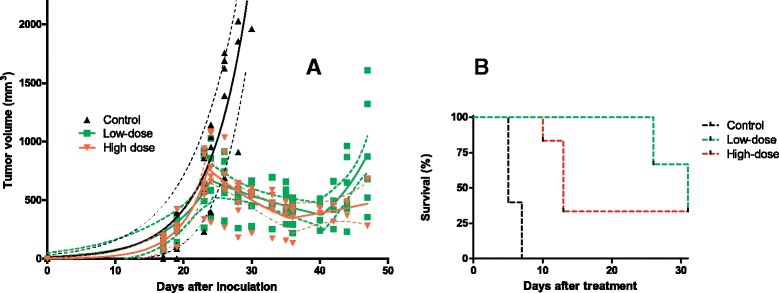
**a** Tumor growth in mice before and after injection of DOTATATE in control animals (4 × 0.3 nmol DOTATATE) and ^213^Bi-DOTATATE at different concentration, in low-dose cohort (16.8 ± 1.3 MBq) and high-dose cohort (33.1 ± 3.7 MBq). Treatment started 23 days after inoculation of AR42J cells. The *solid line* indicates the extrapolated fit calculated for tumor growth, and *dotted lines* indicate the 95% confidence interval for the fit. **b** Survival of AR42J-bearing animals after ^213^Bi-DOTATATE therapy. Low-dose animals received 16.8 ± 1.3 MBq ^213^Bi-DOTATATE, high-dose 33.1 ± 3.7 MBq, and control 4 × 0.3 nmol DOTATATE on 2 consecutive days

Tumor volumes significantly decreased in mice treated with low-dose (16.8 ± 1.3 MBq) and high-dose (33.1 ± 3.7 MBq) ^213^Bi-DOTATATE. Tumor regression was observed in both ^213^Bi-DOTATATE cohorts. Tumor regrowth was delayed until 21 ± 9 days p.i. in animals treated with low-dose ^213^Bi-DOTATATE; see Fig. 4a. One animal in the low-dose showed a weight loss of >15% at day 27 post-treatment. Dramatic weight loss was observed in the high-dose cohort within 14 days post-treatment, necessitating euthanasia of 67% of the animals; see Fig. 4b. Therefore, tumor regrowth doubling time in the high-dose cohort was not determined. The tumor doubling time in the control was 4.0 ± 0.2 days, and both the initial growth and regrowth patterns in the therapy cohorts proceeded with similar doubling times. A median survival of 5 days was found in the control, >30 days in the low-dose, and 13 days in the high-dose; see Fig. 4b. The tumor growth in one animal of the control cohort showed an unusual growth pattern; therefore, the data of this animal was excluded from the calculation of the tumor doubling time and survival analysis.

Animals in the high-dose cohort showed significantly (*P* = <0.05) elevated hemoglobin (Hgb) and hematocrit (Hct) values compared to the low-dose (Hgb (g/dL) 51 ± 7 vs. 39 ± 3 and Hct (%) 16 ± 2 vs. 12 ± 1).

### MTD in nontumor-bearing mice administration w/wo l-lysine

**Fig. 5:**
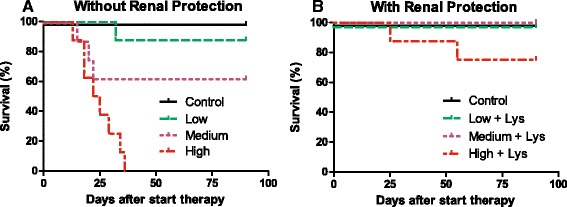
**a**, **b** Survival percentage after ^213^Bi-DOTATATE therapy without (**−**) or with (**+**) l-lysine (35 mg) pre-injection in nontumor-bearing mice; cohort low-dose(−) received cumulatively 13.0 ± 1.6 MBq, low-dose(+) 13.2 ± 0.9 MBq, medium-dose(−) 20.7 ± 0.8 MBq, medium-dose(+) 21.7 ± 1.9 MBq, high-dose(−) 28.7 ± 1.2 MBq, and high-dose(+) 28.3 ± 0.8 MBq (*n* = 8/cohort). Control animals received 4 × 0.3 nmol DOTATATE (*n* = 8)

Renal protection using l-lysine prior to ^213^Bi-DOTATATE administration resulted in prolonged survival for both the medium-dose(+) (21.7 ± 1.9 MBq) and high-dose(+) cohorts (28.3 ± 0.8 MBq), Fig. 5. Medium- and high-dose cohorts without l-lysine showed reduced survival rates compared to medium- and high-dose cohorts pre-treated with l-lysine. No animals in high-dose(−) cohort survived beyond 40 days following treatment. No significant difference in survival was observed following low-dose administration of ^213^Bi-DOTATATE with or without l-lysine (*P* = 0.32) or medium-dose with or without l-lysine (*P* = 0.06). Weight loss was observed in cohorts treated with medium-dose(−), high-dose(−), and high-dose(+) cohorts.

At 90 days post-treatment, all control animals survived. A survival rate of 87.5% was found in the low-dose(−) cohort, 62.5% in the medium-dose(−), and 0% in high-dose(−), Fig. 5a. Cohorts receiving l-lysine pre-treatment, Fig. 5b, a very high survival rate was observed: 100% in the low-dose(+) and 100% in the medium-dose(+). In the high-dose(+) cohort, 75% of the animals survived. A median survival of >90 days was found in control and all cohorts except the high-dose without l-lysine (median survival of 24 days, *P* = 0.0012).

**Fig. 6:**
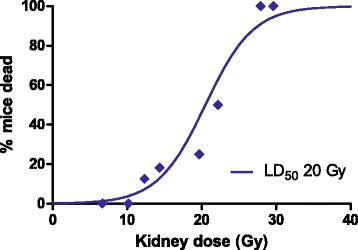
Dose-effect relation between mean renal radiation absorbed dose and the percentage of mice that died before the end of the experiment at 90 days. LD_50_ kidney dose was 20 ± 8 Gy, a threshold LD_5_ = 11 ± 4 Gy (*P* = <0.001). All points represent at least three mice

By integrating the radioactivity over time in the kidney, data obtained from biodistribution study w/wo pre-treatment of l-lysine, a time-integrated activity coefficient (expressed as min/g tissue) of 6.0 ± 2.4 min/g in mice pre-treated with l-lysine and 12.0 ± 3.7 min/g in mice without pre-treatment of l-lysine was found. Based on logistic regression analysis, a LD_50_ of 20 ± 8 Gy was found; see Fig. 6. The number of mice that were euthanized within 90 days was indicated as a function of renal absorbed dose obtained from both biodistribution studies w/wo l-lysine.

**Fig. 7:**
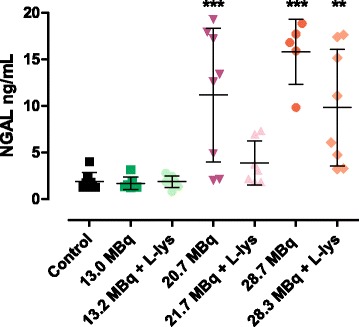
NGAL (ng/mL ± SEM) level in control and ^213^Bi-DOTATATE-treated cohorts with (+) or without (−) pre-treatment of l-lysine (35 mg). Cohort low-dose(−) received cumulatively 13.0 ± 1.6 MBq, low-dose(+) 13.2 ± 0.9 MBq, medium-dose(−) 20.7 ± 0.8 MBq, medium-dose(+) 21.7 ± 1.9 MBq, high-dose(−) 28.7 ± 1.2 MBq, and high-dose(+) 28.3 ± 0.8 MBq. Control animals received 4 × 0.3 nmol DOTATATE. **P value <0.01 and *** *P* value <0.001 versus control values

The highest NGAL level was found in the high-dose(−) cohort, 15.8 ± 3.5 ng/mL. Whereas the NGAL level of control, low-dose cohort w/wo l-lysine was the lowest, 1.9 ± 0.9, 1.9 ± 0.6, and 1.7 ± 0.7 ng/mL, respectively. A significant difference in NGAL level was found in medium-dose(−), high-dose(−), and high-dose(+) vs. control; see Fig. 7.

## Discussion

In this preclinical study, TAT with ^213^Bi-DOTATATE was systematically studied to understand the injected mass of peptide-dependent uptake, radioactivity-related toxicity, and reduction in tumor burden. ^111^In had already been used as a surrogate for ^213^Bi earlier in other preclinical studies [20, 21]. We demonstrated ^111^In is an appropriate surrogate radionuclide for in vivo preclinical studies of PK in tumors allowing the results obtained from ^111^In to be used for ^213^Bi-dosimetry calculation. Both ^213^Bi and ^111^In form highly stable complexes with DOTA-somatostatin analogs, including DOTATATE, and show similar affinities for SSTR_2_ in tumor.

For PRRT, it is essential to determine the optimal injected mass of radioligand by defining PK of radiopeptides in animal models, given that the injected mass of radioligand influences tumor uptake, the resultant radiation absorbed dose, and eventually the efficacy of the therapy. Moreover, increasing the injected mass of radioligand can diminish the pharmacological selectivity by binding to other SSTR-positive organs [22], which is not beneficial in the case of TAT and may cause off-target toxicities. The optimal injected mass of ^111^In-OctreoScan® to obtain the best signal to background ratio for tumor versus other organs was reported as 0.07 nmol in mice (3.5 pmol/g mice) [23]. De Jong et al. showed a “bell-shape” curve for dependent tumor uptake in AR42J tumor-bearing rats as a function of injected mass of peptide, where 0.4 nmol ^111^In-DOTATOC (1.8 pmol/g rat) gave the maximum tumor uptake [24].

In this study, the highest absorbed tumor dose (0.66 Gy/MBq) was found at injected mass of peptide of 0.03 nmol (1.07 pmol/g mice). However, lower and more practical specific activity (MBq/nmol) ^213^Bi-DOTATATE, 0.3 nmol (10.7 pmol/g mice) was chosen for the administration in this study allowing sufficient tumor uptake to realize therapeutic effects. A similar mean radiation absorbed dose of ^213^Bi-DOTATATE was determined for 0.1 and 0.3 nmol injected mass of peptide, as the tumor uptakes as function of time of both 0.1 and 0.3 nmol peptide were similar.

^111^In-DOTATATE is not an appropriate surrogate of ^213^Bi-DOTATATE to determine renal uptake as a significant difference was observed at 60 min p.i. between ^111^In-DOTATATE and ^213^Bi-DOTATATE. With an absence of SSTR_2_ receptors in the kidney, the high renal uptake is not related to SSTR expression. The renal uptake of the labeled peptide is thought to be influenced by the difference in the electrostatic charge of DOTA complex with ^111^In and ^213^Bi [9, 10, 25], leading to different interactions with megalin or cubilin [26]. Furthermore, ^213^Bi^3+^ is known to bind strongly to metallothionein in the kidneys [27], which might lead to a high renal uptake. Apart from high renal uptake, a significantly higher uptake was also found in the pituitary and a higher radioactivity level in plasma. The pituitary gland is a very small organ. During organ harvesting, a systematic uncertainty is introduced by the chance to include surrounding tissue in the weight used for the uptake per gram calculation, resulting to an under- or overestimation of pituitary uptake, which might explain our findings. The high renal uptake and slow clearance rate of ^213^Bi-DOTATATE indicates tubular reabsorption of ^213^Bi-DOTATATE; this might be the cause of higher radioactivity in plasma as well. ^111^In-DOTATATE showed a slightly significantly higher uptake than ^213^Bi-DOTATATE in pancreas tissue, as yet we do not have an explanation for this difference.

In this study, we were not able to examine the differences in PKs of these radiopharmaceuticals in pituitary, plasma, and plasma, due to the small number of animal per group and limited time points.

Despite the differences in PK profiles of ^111^In-DOTATATE and ^213^Bi-DOTATATE in some organs and tissues, ^111^In-DOTATATE still showed to be a proper substitute for tumor uptake, since the PK profile of the tumor uptake was similar to that of ^213^Bi-DOTATATE. However, the use of a surrogate radionuclide should be carefully chosen, since each alternate radionuclide has limitations.

Weight loss in animals is often an indicator of toxicity, and the most radiosensitive organs for PRRT are the bone marrow and kidney [8, 28, 29]. In this study, we observed severe weight loss in 67% of animals exposed to high-dose ^213^Bi-DOTATATE (cumulative 33.1 ± 3.7 MBq), within 2 weeks after treatment, indicating acute toxicity. This might be explained by the high renal uptake resulting in a high renal absorbed dose, which increased the risk of acute nephrotoxicity due to limited sublethal damage tissue repair. To investigate acute renal toxicity, a short-term toxicity study over 90 days was performed instead of a follow-up period over 6–12 months, which is commonly performed to investigate long-term chronic nephrotoxicity. A significant reduction of renal activity (50%) was found in animals pre-treated with l-lysine in this study. Our findings indicate that pre-treatment with l-lysine improved survival of animals receiving medium- and high-dose ^213^Bi-DOTATATE resulting from the reduction of renal activity. Song et al. showed in their study a threefold reduction in renal activity following lysine pre-treatment [13]. This result differs significantly from our findings but might be attributed to their method of lysine application used during the therapy procedures, rather than immediately prior. Kobayashi et al. demonstrated that the kidney uptake was influenced by the timing of l-lysine administration [30], such that renal blocking by l-lysine was maximized when i.p. administration of l-lysine was given immediately before administration of the radiolabeled of anti-Tac murine MoAb fragment. In our study, we have chosen to start the therapy 2–10 min after i.p. administration of l-lysine to protect the kidneys, since DOTATATE is a relative small molecule and rapidly cleared from the blood. Radioactivity in the blood or uptake of the bone marrow is generally used as an indicator for myelotoxicity. Pre-administration of l-lysine did not significantly affect the radioactivity measured in neither whole blood nor femur uptake in tumor-bearing mice. The mean radiation absorbed dose for whole blood and femur (see Additional file 1) was 3.3 and 1.3 Gy in mice with pre-treatment of l-lysine in the high-dose cohort, whereas without l-lysine, these values were 2.7 and 1.0 Gy. These absorbed doses were lower than the MTD of 25 MBq ^213^Bi-DOTA-AMBA in PC3-tumor-bearing mice, corresponding to the MTD at a mean absorbed dose of 4 Gy in the blood [31]. Therefore, we concluded the bone marrow is not a limiting organ in our study.

The LD_50_ found for the renal absorbed dose was 20 Gy in this study. Acute renal toxicity at 100–140 Gy was reported by Behr et al., after administration of ^90^Y-fab fragments, leading to death of all mice within 2–3 weeks [32]. This corresponds to our observation in cohorts after high-dose ^213^Bi-DOTATATE administration, with more than 90% of the animals dead at radiation absorbed dose >28 Gy. Hence, the relative biological effect (RBE) was 4–5 for acute renal toxicity, leading to death within 2–3 weeks, when comparing the absorbed doses in both studies. This estimate for the RBE for ^213^Bi-DOTATATE appears to be comparable to the RBE value of 4 used for delayed renal toxicity by Song et al. [13]. Specific uptake in functional units of the kidney might cause changes in radiation absorbed doses to radiation-sensitive structures like the glomeruli that could result in less damage than predicted from whole-organ radiation dosimetry. Small-scale micro-dosimetry using the sub-organ model of Hobbs et al. [33] indicates a possible lowering of the absorbed dose to the glomeruli when ^213^Bi is taken up in the proximal tubules by 44% in comparison to homogeneous uptake in the mouse cortex assuming equal kinetics. We found no indication for this sparing effect; otherwise, the RBE would be in the order of 6–8. A direct comparative study would be needed to determine both the RBE and the PK of ^213^Bi- and ^90^Y- or ^177^Lu-labeled peptides inside the kidneys and its functional units.

In this pilot study, NGAL was used as a biomarker to evaluate late-stage renal changes after therapy. NGAL is sensitive to acute kidney injury (AKI) for detection of renal functions in early nephrotoxicity state [16, 34]. No nephrotoxicity was found in the low-dose(−) and low-dose(+) cohorts, corresponding to another study done using similar injected mass of radioactivity (MBq) ^213^Bi-DOTATATE as TAT in nude mice in two different tumor models wherein nephrotoxicity was investigated by ^99m^Tc-DMSA as a kidney marker [35]. Overall, NGAL levels were lower in mice pre-treated with l-lysine than mice without pre-treatment at similar dose of ^213^Bi-DOTATATE. However, no significant difference was found between those cohorts, which might be explained since NGAL was measured day 90 after TAT, whereby some repair and recovery of the kidney might already occur. Furthermore, the mean renal absorbed dose for the medium-dose(−) and the high-dose(+) cohorts was 23 and 16 Gy, respectively. These absorbed doses were similar to the calculated renal LD_50_, at which 50% of the treated animals would develop acute nephrotoxicity. The sigmoid dose-effect curve for renal toxicity (Fig. 6) shows a steep slope, contributing to a great variation in NGAL values observed at absorbed doses just above and below the LD_50_ value. In this study, NGAL proved to be a valuable tool to examine AKI for TAT using ^213^Bi as radionuclide supporting its use in future investigations of nephrotoxicity caused by ^213^Bi. The use of NGAL as a biomarker of nephrotoxicity is feasible and cost-effective compared to conventional approaches to determine renal functionality in preclinical studies. Creatinine, the most commonly used parameter to determine kidney injury, lacks the ability to evaluate kidney injury at early stages following PRRT. ^99m^Tc-MAG3 and ^99m^Tc-DMSA for preclinical applications are invasive, by the use of high radioactivity for imaging, and require additional data extraction and analysis. In addition to NGAL, kidney injury molecule-1 (KIM-1) and cystatine-C are promising biomarkers for both acute and chronic kidney disease [15]. The ability to study early and late kidney injury is essential in TAT, using a combination of both conventional methods, and these commercially available biomarkers could provide more information leading to more understanding of the underlying mechanisms involved in kidney injury after TAT.

## Conclusions

^213^Bi-DOTATATE showed therapeutic effects to reduce tumor size and prolong survival. Potential nephrotoxicity caused by ^213^Bi-DOTATATE was overcome by pre-treatment with l-lysine. ^213^Bi-DOTATATE with l-lysine pre-treatment shows promise as TAT of SSTR_2_-expressing tumors. The biomarker NGAL offers a new approach to study nephrotoxicity following TAT.

## Additional file

Additional file 1: Improved safety and efficacy of ^213^Bi-DOTATATE targeted alpha therapy of somatostatin receptor-expressing neuroendrocrine tumors in mice pre-treated with L-lysine. (DOCX 31 kb)
