# Supplementary material for: Improved safety and efficacy of 213Bi-DOTATATE-targeted alpha therapy of somatostatin receptor-expressing neuroendocrine tumors in mice pre-treated with l-lysine
Source: EJNMMI Res. 2016 Nov 21;6:83. doi: 10.1186/s13550-016-0240-5 (PMC5118228; doi:10.1186/s13550-016-0240-5)
Supplement: Additional file 1: — Improved safety and efficacy of 213Bi-DOTATATE targeted alpha therapy of somatostatin receptor-expressing neuroendrocrine tumors in mice pre-treated with L-lysine. (DOCX 31 kb) [file 13550_2016_240_MOESM1_ESM.docx]

**Additional file**

**Results:**

**Table S2**

| **^111^In-DOTATATE (0.03 nmol) uptake (%IA/g) in organs as function of time post injection** | | | | |
| --- | --- | --- | --- | --- |
| **Organs** | **3 min** | **10 min** | **30 min** | **60 min** |
| **Tumor** | 6.9±3.6 | 10.7±2.5 | 15.1±7.5 | 12.8±6.7 |
| **Muscles** | 1.2±0.7 | 0.9±0.3 | 0.3±0.1 | 0.07±<0.05 |
| **Pancreas** | 6.0±1.5 | 1.6±1.4 | 4.7±2.7 | 9.2±1.9 |
| **Stomach** | 5.6±0.7 | 7.9±2.2 | 8.1±1.3 | 7.6±1.1 |
| **Kidney** | 19.2±8.4 | 16.0±4.1 | 14.0±4.1 | 12.3±3.7 |
| **Pituitary** | 1.0±1.7 | 0.2±0.2 | 0.5±0.8 | <0.05 |
| **Red blood cell** | 3.5±2.5 | 1.7±0.5 | 0.6±0.2 | 0.3±0.08 |
| **Plasma** | 13.3±1.6 | 8.2±1.6 | 3.2±1.0 | 0.8±0.1 |
| **Adrenals** | 2.7±1.6 | 3.4±2.3 | 4.7±4.4 | 4.6±5.6 |

**Table S2**, Biodistribution data in AR42J tumor-bearing nude mice. Uptake at 3, 10, 30 and 60 min p.i. and expressed in percentage injected activity per gram tissue (%IA/g) after ^111^In-DOTATATE (0.03 nmol, 1.07 pmol/g ) administration (n=4/cohort).

**Table S3**

| **^111^In-DOTATATE (0.1 nmol) uptake (%IA/g) in organs as function of time post injection** | | | | |
| --- | --- | --- | --- | --- |
| **Organs** | **3 min** | **10 min** | **30 min** | **60 min** |
| **Tumor** | 2.8±1.8 | 11.8±3.6 | 10.9±3.6 | 9.9±2.6 |
| **Muscles** | 1.5±0.6 | 0.7±0.2 | 0.4±0.4 | 0.3±0.4 |
| **Pancreas** | 11.9±4.0 | 9.9±4.2 | 7.0±1.4 | 4.7±0.5 |
| **Stomach** | 4.4±1.1 | 5.2±1.1 | 6.3±2.0 | 6.0±1.8 |
| **Kidney** | 21.6±3.9 | 15.6±3.1 | 16.1±5.5 | 16.7±8.6 |
| **Pituitary** | 0.3±0.2 | 0.2±0.09 | 0.1±0.2 | <0.05 |
| **Red blood cell** | 5.2±0.7 | 1.7±0.5 | 0.8±0.4 | 0.2±0.1 |
| **Plasma** | 13.2±1.7 | 8.6±1.4 | 3.4±1.7 | 1.0±0.6 |
| **Adrenals** | 2.1±0.6 | 1.9±0.3 | 2.1±0.7 | 1.3±0.2 |

**Table S3**, Biodistribution data in AR42J tumor-bearing nude mice. Uptake at 3, 10, 30 and 60 min p.i. and expressed in percentage injected activity per gram tissue (%IA/g) after ^111^In-DOTATATE (0.1 nmol, 3.6 pmol/g mice) administration (n=4/cohort).

**Table S4**

| **^111^In-DOTATATE (0.3 nmol) uptake (%IA/g) in organs as function of time post injection** | | | | |
| --- | --- | --- | --- | --- |
| **Organs** | **3 min** | **10 min** | **30 min** | **60 min** |
| **Tumor** | 4.9±2.2 | 8.0±1.2 | 9.9±3.0 | 9.9±0.7 |
| **Muscles** | 1.1±0.1 | 0.5±0.1 | 0.3±0.1 | <0.05 |
| **Pancreas** | 4.9±0.7 | 3.5±0.3 | 3.2±0.4 | 3.0±1.0 |
| **Stomach** | 4.5±0.8 | 3.9±0.4 | 3.5±0.2 | 3.4±0.8 |
| **Kidney** | 17.2±5.4 | 12.8±2.2 | 16.9±6.3 | 13.4±2.8 |
| **Pituitary** | 0.3±<0.05 | 0.2±0.05 | 0.1±<0.05 | <0.05 |
| **Red blood cell** | 2.6±0.6 | 1.3±0.4 | 0.6±0.1 | <0.05 |
| **Plasma** | 13.4±1.4 | 7.2±2.0 | 3.4±0.5 | 0.7±0.2 |
| **Adrenals** | 2.3±0.6 | 1.2±0.4 | 1.0±0.1 | 0.8±0.3 |

**Table S4**, Biodistribution data in AR42J tumor-bearing nude mice. Uptake at 3, 10, 30 and 60 min p.i. and expressed in percentage injected activity per gram tissue (%IA/g)after ^111^In-DOTATATE (0.3 nmol, 10.7 pmol/g mice) administration (n=4/cohort).

**Table S5**

| **^111^In-DOTATATE (1 nmol) uptake (%IA/g) in organs as function of time post injection** | | | | |
| --- | --- | --- | --- | --- |
| **Organs** | **3 min** | **10 min** | **30 min** | **60 min** |
| **Tumor** | 5.6±2.0 | 6.7±2.9 | 2.9±0.6 | 7.0±1.1 |
| **Muscles** | 1.2±0.4 | 1.0±0.5 | 0.2±0.09 | <0.05 |
| **Pancreas** | 3.5±2.4 | 1.9±1.0 | 0.7±0.2 | 1.5±0.3 |
| **Stomach** | 2.4±1.1 | 1.7±0.6 | 0.8±0.2 | 1.8±0.6 |
| **Kidney** | 17.5±9.7 | 14.2±7.0 | 6.0±1.8 | 14.1±3.4 |
| **Pituitary** | 0.1±0.09 | 0.8±1.4 | <0.05 | <0.05 |
| **Red blood cell** | 2.3±0.4 | 1.3±0.5 | 0.3±0.1 | 0.4±0.1 |
| **Plasma** | 11.3±4.9 | 7.6±3.1 | 1.4±0.5 | 0.6±0.2 |
| **Adrenals** | 1.4±0.7 | 1.8±1.1 | 0.5±0.09 | 0.5±0.05 |

**Table S5**, Biodistribution data in AR42J tumor-bearing nude mice. Uptake at 3, 10, 30 and 60 min p.i. and expressed in percentage injected activity per gram tissue (%IA/g)after ^111^In-DOTATATE (1 nmol, 35.7 pmol/g mice) administration (n=4/cohort).

**Table S6**

| **^111^In-DOTATATE (3 nmol) uptake (%IA/g) in organs as function of time post injection** | | | | |
| --- | --- | --- | --- | --- |
| **Organs** | **3 min** | **10 min** | **30 min** | **60 min** |
| **Tumor** | 2.9±1.1 | 3.8±0.3 | 3.6±0.2 | 3.3±0.3 |
| **Muscles** | 1.3±0.3 | 0.8±0.3 | 0.3±0.1 | <0.05 |
| **Pancreas** | 2.2±0.4 | 1.6±0.2 | 1.0±0.2 | 0.8±1.0 |
| **Stomach** | 3.2±0.5 | 2.3±0.2 | 1.5±0.6 | 1.6±0.7 |
| **Kidney** | 19.1±3.1 | 14.6±2.9 | 14.5±1.1 | 14.7±2.1 |
| **Pituitary** | 0.2±0.06 | 0.1±0.06 | 0.08±0.06 | <0.05 |
| **Red blood cell** | 4.3±1.9 | 2.4±0.3 | 0.6±0.2 | <0.05 |
| **Plasma** | 13.8±1.7 | 8.4±0.9 | 2.8±0.7 | 0.6±0.2 |
| **Adrenals** | 1.9±0.7 | 1.1±0.7 | 0.6±0.2 | 0.3±0.06 |

**Table S6**, Biodistribution data in AR42J tumor-bearing nude mice. Uptake at 3, 10, 30 and 60 min p.i. and expressed in %IA/g after ^111^In-DOTATATE (3 nmol, 107 pmol/g mice) administration (n=4/cohort).

**Table S7**

| **Radiation absorbed dose per administered activity (Gy/MBq) with or without L-lysine** | | | | | | | | | | | | | | |
| --- | --- | --- | --- | --- | --- | --- | --- | --- | --- | --- | --- | --- | --- | --- |
| **Organs** | **With L-lysine** | | | | | | | **Without L-lysine** | | | | | | |
|  | **^213^Bi**  **α β** | | **^213^Po**  **α** | **^209^Tl**  **β + γ** | **^209^Pb**  **β** | **Total**  **α β** | | **^213^Bi**  **α β** | | **^213^Po**  **α** | **^209^Tl**  **β + γ** | **^209^Pb**  **β** | **Total**  **α β** | |
| **Tumor** | 9.20E-03 | 2.87E-02 | 6.05E-01 | 8.60E-04 | 1.41E-02 | **6.14E-01** | **4.40E-02** | 9.90E-03 | 3.14E-02 | 6.51E-01 | 9.60E-04 | 1.58E-02 | **6.61E-01** | **4.80E-02** |
| **Pancreas** | 2.00E-03 | 4.50E-03 | 1.34E-01 | 1.00E-04 | 8.90E-04 | **1.36E-01** | **5.00E-03** | 2.00E-03 | 4.50E-03 | 1.32E-01 | 1.00E-04 | 7.00E-04 | **1.34E-01** | **5.00E-03** |
| **Stomach** | 9.00E-04 | 2.60E-03 | 6.00E-02 | 7.00E-05 | 2.20E-04 | **6.10E-02** | **3.00E-03** | 1.30E-03 | 3.90E-03 | 8.84E-02 | 1.10E-04 | 5.90E-04 | **9.00E-02** | **5.00E-03** |
| **Kidney** | 8.00E-03 | 2.37E-02 | 5.25E-01 | 6.30E-04 | 1.40E-03 | **5.33E-01** | **2.60E-02** | 1.61E-02 | 4.71E-02 | 1.06E+00 | 1.30E-03 | 6.20E-03 | **1.07E+00** | **5.50E-02** |
| **Adrenals** | 8.00E-04 | 1.80E-03 | 5.49E-02 | 4.00E-05 | 1.30E-04 | **5.60E-02** | **2.00E-03** | 5.00E-04 | 1.50E-03 | 3.56E-02 | 2.00E-05 | 2.00E-04 | **3.60E-02** | **2.00E-03** |
| **Femur** | 6.00E-04 | 1.60E-03 | 4.13E-02 | 4.00E-05 | 1.00E-04 | **4.20E-02** | **2.00E-03** | 5.00E-04 | 1.30E-03 | 3.16E-02 | 3.00E-05 | 1.00E-04 | **3.20E-02** | **1.00E-03** |
| **Blood** | 1.60E-03 | 5.20E-03 | 1.08E-01 | 1.40E-04 | 2.00E-04 | **1.09E-01** | **6.00E-03** | 1.30E-03 | 4.30E-03 | 8.80E-02 | 1.20E-04 | 1.60E-04 | **9.00E-02** | **5.00E-03** |

**Table S7,** Calculated total radiation absorbed dose after ^213^Bi-DOTATATE (in Gy/MBq) with or without L-lysine (35 mg) in tumor-bearing mice, including the radiation dose caused by ^213^Bi daughters in different organs, the contribution to the dose by α-particles and by β-particles (+γ) is indicated separately.
